# Reading Performance Is Enhanced by Visual Texture Discrimination Training in Chinese-Speaking Children with Developmental Dyslexia

**DOI:** 10.1371/journal.pone.0108274

**Published:** 2014-09-23

**Authors:** Xiangzhi Meng, Ou Lin, Fang Wang, Yuzheng Jiang, Yan Song

**Affiliations:** 1 Department of Psychology, Peking University, Beijing, China; 2 State Key Laboratory of Cognitive Neuroscience and Learning & IDG/McGovern Institute for Brain Research, Beijing Normal University, Beijing, China; 3 School of languages and communication, Beijing Jiaotong University, Beijing, China; 4 Center for Collaboration and Innovation in Brain and Learning Sciences, Beijing Normal University, Beijing, China; 5 The Joint PekingU-PolyU Center for Child Development and Learning, Peking University, Beijing, China; University of Pennsylvania, United States of America

## Abstract

**Background:**

High order cognitive processing and learning, such as reading, interact with lower-level sensory processing and learning. Previous studies have reported that visual perceptual training enlarges visual span and, consequently, improves reading speed in young and old people with amblyopia. Recently, a visual perceptual training study in Chinese-speaking children with dyslexia found that the visual texture discrimination thresholds of these children in visual perceptual training significantly correlated with their performance in Chinese character recognition, suggesting that deficits in visual perceptual processing/learning might partly underpin the difficulty in reading Chinese.

**Methodology/Principal Findings:**

To further clarify whether visual perceptual training improves the measures of reading performance, eighteen children with dyslexia and eighteen typically developed readers that were age- and IQ-matched completed a series of reading measures before and after visual texture discrimination task (TDT) training. Prior to the TDT training, each group of children was split into two equivalent training and non-training groups in terms of all reading measures, IQ, and TDT. The results revealed that the discrimination threshold SOAs of TDT were significantly higher for the children with dyslexia than for the control children before training. Interestingly, training significantly decreased the discrimination threshold SOAs of TDT for both the typically developed readers and the children with dyslexia. More importantly, the training group with dyslexia exhibited significant enhancement in reading fluency, while the non-training group with dyslexia did not show this improvement. Additional follow-up tests showed that the improvement in reading fluency is a long-lasting effect and could be maintained for up to two months in the training group with dyslexia.

**Conclusion/Significance:**

These results suggest that basic visual perceptual processing/learning and reading ability in Chinese might at least partially rely on overlapping mechanisms.

## Introduction

Developmental dyslexia is a disorder characterized by difficulty in learning to read without lack of motivation, educational opportunities or intellectual insufficiency [1]. The root cause of dyslexia remains controversial. In past decades, two major frameworks were established to explain the origin, mechanisms and symptoms of developmental dyslexia. The first framework, from a linguistic point of view, hypothesizes a core deficit in accessing and/or manipulating phonemic information in children and adults with developmental dyslexia [2], [3]. The second, from a nonlinguistic perspective, posits that the phonological deficit occurring on the linguistic level may stem from more fundamental deficits in information processing within the sensory domain, such as acoustic-auditory information processing, auditory temporal processing [4]–[7], and/or visual processing [8], [9].

Accordingly, the remediation of dyslexia has also followed these two lines: the linguistic/phonological and the nonlinguistic orientated treatments. Aside from a large number of studies showing the remedial effect of linguistic programs, mostly targeted at improving phonological skills [10]–[12] and reading skills in developmental dyslexia, an increasing number of studies have reported that nonlinguistic intervention programs also substantially improve reading performance in individuals with dyslexia. These nonlinguistic training methods include auditory temporal training/musical training [5], [13]–[16], visual programs aimed at improving the function of the magnocellular system [17] or alleviating the crowding phenomenon by increasing letter spacing [18], and action video games that significantly improve reading speed in individuals with dyslexia by efficiently improving attention abilities [19]. The effects of these nonlinguistic intervention protocols suggest that training the perceptual and cognitive skills underpinning poor phonological development and/or poor reading would be beneficial to reading and phonological development [15].

Indeed, studies directly involving visual perceptual learning did reveal a positive effect on reading speed. Perceptual learning is evidenced by an improvement of perceptual performance as a function of training [20], which has been shown to enlarge visual span size and produce corresponding improvement in reading speed in young children [21] and older adults [22]. The texture discrimination task (TDT) is one of the most intensively studied PL tasks. In this task, a texture stimulus is first presented for a short time, followed by a blank interval (stimulus-to-mask onset asynchrony, SOA), and then by the mask. The subjects need to decide whether the fixation letter was “T” or “L”, and then indicate the texture target orientation (horizontal or vertical). Behavioral performance on both tasks was measured as the proportion of correct responses for different SOAs. Our recent behavioral study reported that Chinese-speaking children with dyslexia exhibited visual perceptual learning deficits [23]. In this study, when all of the participants were asked to perform the classical TDT with the SOA at 300 ms, the SOA for reaching 80% accuracy did not show improvement over 5 days of training in the children with dyslexia while this SOA steadily decreased over the training sessions in the control group. When an adaptive procedure was used to determine the SOA for each participant during training, both the children with dyslexia and the control group attained perceptual learning; more importantly, over individual participants, the SOA was negatively correlated with their performance in Chinese character recognition. These findings suggest that deficits in visual perceptual processing and learning might, in part, underpin the reading difficulty in Chinese.

In the current study, we further examined whether visual perceptual learning has a positive effect in improving the performance of reading. Before and after 10 sessions of TDT training, a series of reading measures were performed in two groups of children (dyslexia children and typically developed control children). This study can provide a more detailed picture of the relationship between visual perceptual learning and developmental dyslexia. Of particular empirical importance, if visual perceptual training can benefit the development of reading skills in children with dyslexia, this finding will have significant implications for the prevention and diagnosis and the design of educational curricula for developmental dyslexia.

## Materials and Methods

### Ethics Statement

All experimental procedures were approved by the Ethics Committee of the Department of Psychology, Peking University. The research was conducted according to the principles expressed in the Declaration of Helsinki. Written consent was obtained from all of the children as well as their parents. All experiments were performed at the joint PekingU-PolyU center for Child Development and Learning of Peking University.

### Participants

Thirty-six Chinese-speaking children, eighteen with dyslexia, eighteen with typical readers, in grades four, five, and six, were recruited to participate in this study according to the procedures described below. None of the participants had a history of neurological diseases or psychiatric disorders. In particular, the DSM-IV Attention-Deficit/Hyperactivity Disorder (ADHD) Scale [24] was used to exclude children with ADHD. All of the participants were right-handed and had normal or corrected-to-normal vision. Informed consent was obtained from each participant and their parents.

The children were placed in the dyslexia group if their scores on the character recognition test were at least 1.5 grades below the norm (below), and if their reading fluency test scores were lower than the mean scores for their grades (below). Additionally, they had typically developed IQ scores, as assessed by *Raven’s Standard Progressive Matrices* [25]. The chronological age- and IQ -matched control children were selected from among their peers (Table 1). Similar procedures for recruiting children with dyslexia or with reading impairment were implemented by previous studies [26].

**Table 1:** Characteristics of the participants, with standard deviation in parenthesis.

	Group with dyslexia	Control group	t	P
**N**	18	18		
**Gender/Boys**	15	11		
**Age (years)**	10.34 (0.78)	10.41 (0.74)	0.294	NS
**Raven (Percentile)**	69 (17.17)	75 (18.11)	1.039	NS
**Reading fluency (Mean)**	28.06 (5.87)	49.56 (9.01)	8.479	<.001
**Vocabulary (Mean)**	2446.8 (254.57)	3054.1 (182.61)	8.224	<.001

For age, the numbers are mean years for the dyslexia and control groups. For the Raven, the numbers are mean percentiles for the dyslexia and control groups. For reading fluency, the numbers represent means of items that the dyslexia and control groups answered correctly. For Chinese character recognition, the numbers are the numbers of characters children could use correctly in word composition.

Prior to the TDT training, each group of children was split into two equivalent training and non-training groups on the performance of all of the reading measures, IQ, and TDT (Table 2).

**Table 2:** Characteristics of training and non-training groups of participants, with standard deviation in parenthesis.

	Group with Dyslexia			Control group		
	Training group	Non-traininggroup	*p*	Traininggroup	Non-traininggroup	*p*
**N**	9	9	n.s.	9	9	n.s.
**Age (years)**	10.5 (0.7)	10.2 (0.9)	n.s.	10.4 (0.8)	10.4 (0.7)	n.s.
**Raven (Percentile)**	68.9 (15.6)	69.4 (19.6)	n.s.	75 (20.3)	75.6 (16.8)	n.s.
**TDT threshold SOAs** **of Pre-test (ms)**	555 (297)	615 (289)	n.s.	377 (197)	451 (215)	n.s.
**Reading fluency**	26.3 (4.2)	29.8 (6.9)	n.s.	51.4 (10.5)	47.6 (7.4)	n.s.
**Vocabulary**	2454.7 (306)	2438.8 (209)	n.s.	3065.4 (135)	3042.7 (228)	n.s.

For age, the numbers are mean years for training and non-training groups with dyslexia and control. For the Raven, the numbers are mean percentiles for each group of participants. For reading fluency, the numbers represent means of items that each group of participants answered correctly. For Chinese character recognition, the numbers are the numbers of characters children could use correctly in word composition.

### Linguistic tests

Two reading measures, character recognition accuracy and reading fluency, were utilized to assess the participants’ reading abilities, and to investigate the relationship between visual perceptual processing/learning and the accuracy and fluency of reading skills. The reason for using two reading measures was that a review of the literature revealed that both accuracy and fluency are important for reading development and that both are impaired in children with dyslexia [27], [28]. Additionally, only a few studies have directly addressed the association between reading measures and visual perceptual processing/learning [21]–[23]. Therefore, we also used two reading measures to classify which specific reading skills (recognition accuracy or the speed) are associated with the visual perceptual processing/learning ability and which are enhanced by visual perceptual training. This study will probably permit us to infer what psychological processes are involved in visual perceptual learning.

*The Standardized Chinese Character Recognition Test* [29] consists of 210 characters, and is divided into ten groups based on the reading-difficulty level. The participants were asked to write down a compound word based on a constituent morpheme provided on the sheet. Their performance was measured by the total number of correct characters (morphemes) that they could utilize in word compositions. The participants had to know morpheme combination rules to form a compound word. The scores from this test formed the index of the participants’ Chinese character recognition performance.

In this standardized Chinese character recognition test [29], the medium difficulty characters with a high degree of differentiation were stratified, sampling from the character corpus of primary school textbooks for each grade. Then, 5102 children from grades 1–5 were tested with the tests for their own grades. The mean and standard deviation of the performance of character recognition were calculated for each grade. The standard Z scores for each grade were then obtained with (X_raw score_−Mean_certain grade_)/standard deviation_certain grade_ as the norm. The total number of correct characters each participant completed corresponds to a specific reading grade through the norm. For example, if a child was in fifth grade when tested and the total number of characters completed correctly corresponded to third grade on the norm, he/she would be identified as a participant with dyslexia.

*The Reading Fluency Test* was composed of 95 sentences [30]. Each sentence was paired with five multiple-choice pictures. The participants were asked to read each sentence and select, from five pictures, the one that best illustrated the meaning of the sentence. For instance, for the sentence “She is playing with a ball in front of the house”, if a participant circles the picture of a girl playing a ball in front of a house, the answer is correct. If the participant circles pictures in which three or two children are playing ball in the yard or in the house, the answers are incorrect. The children were encouraged to complete as many paragraphs as possible within a ten-minute time period. The total number of sentences that the participants could understand determined the performance score. This task required the rapid retrieval and retention of lexical information and construction of sentential representation. All of the sentences in this test are composed of high frequency characters and words; therefore, it is very easy for participants to grasp the meaning of each sentence. We used this test to evaluate children’s reading speed by the total number of sentences they can accomplish. Because participants in this study were recruited from grades four, five, and six, each participant’s reading fluency score was compared to the mean score of the grade he/she belonged to. The mean score was calculated from the total number of correct answers the grade children completed, divided by the total number of children participating from that grade.

Additionally, *Raven’s Standard Progressive Matrices* were used to measure the children’s nonverbal Intelligence Quotient (IQ). The scoring procedures were based on the Chinese norm [25].

### Visual perceptual learning test and training

#### Stimuli

A texture discrimination task (TDT) was employed that has been used in a number of visual perceptual learning studies [31]–[33]. The stimuli, white (54 cd/m^2^) on a uniform black background, were displayed on a 21-inch gamma linearized CRT monitor (1024×768 pixels at 85 Hz) at a 110 cm viewing distance. All of the stimuli were generated by a MATLAB program. All of the experiments were performed using E-prime (1.0) software.

**Figure 1:**
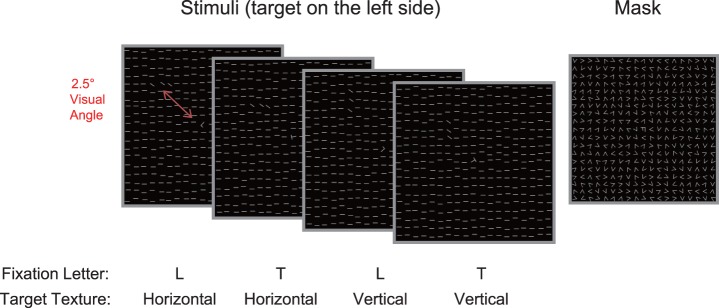
Experimental stimuli displays. The texture stimulus (left) was composed of three adjacent diagonal bars (vertically or horizontally aligned) embedded within a background of horizontal bars, with a letter “T” or “L” as the fixation. The mask (right) was made of randomly oriented “V”, with superimposed “T” and “L” at the fixation position.

The visual stimulus was a texture display made of 19×19 high-contrast horizontal line segments positioned in the central visual field, covering an area of a 14°×14° visual angle (Figure 1). The lines were .46°×.04° in size and spaced .76° apart. The position of each line segment was jittered randomly by 0°∼.1°. A randomly rotated letter “T” or “L” was presented at the bottom of the texture stimulus for subjects to fixate. A target was generated by tilting three adjacent bars in the texture stimuli from horizontal to 135°, forming either a horizontal (Figure 1) or a vertical configuration. The target array was embedded either in the upper left or upper right quadrant of the visual field at a location of 2.5°∼5° away from the fixation. A mask of the same size as the stimulus was made of 19×19 randomly oriented V-shaped patterns except at the fixation, where a superimposed “T” and “L” were used to mask the “T” or “L” in the stimulus pattern (Figure 1).

#### Procedure

**Figure 2:**
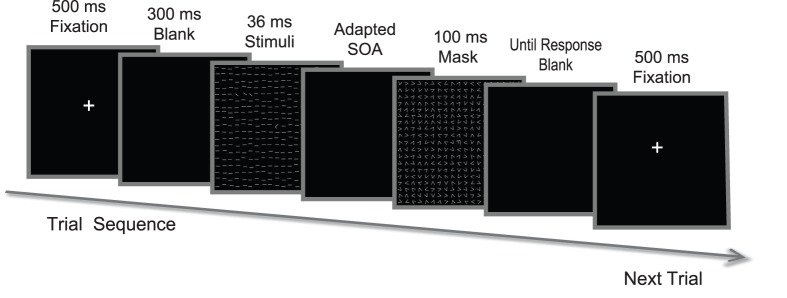
Trial sequences of texture discrimination task.

During each test trial, observers first fixated for 500 ms on a small central cross, where the fixation letter (“T” or “L”) was displayed (Figure 2). Subsequent to a 300 ms blank, the texture stimulus was presented for 36 ms, followed by a blank interval (stimulus-to-mask onset asynchrony, SOA), and then by the mask for 100 ms. The central cross remained on the CRT until the observers gave their responses, first reporting the fixation letter (“T” or “L”) and then indicating the texture target orientation (horizontal or vertical). No feedback was given. The response was deemed correct when judgments on both the letter and the target texture were correct.

The staircase followed a 2-down-1-up rule, which resulted in a 66.7% convergence rate. Each staircase (approximately 60 trials) consisted of four preliminary reversals and six experimental reversals. The geometric mean of the experimental reversals was taken as the threshold for each staircase. There were three staircases in the test sessions (pre- and post-test, approximately 20 minutes) and six staircases in the training sessions (approximately 50 minutes). For all of the participants, the step-size was 36 ms until the fourth reversal and then changed to 24 ms. During the training, the horizontal or vertical target array was always presented at a fixed location (either in the upper-left or upper-right visual-field quadrant, “the trained location”), which was counterbalanced across participants.

To ensure that the children understood how to perform the task, all of the participants practiced the task without mask at the beginning of the experiment. First, the children were told only to do the central letter discrimination task and to ignore the target texture task (40 trials). Then, they were asked to process the central letter and peripheral stimuli simultaneously (40 trials). Finally, they practiced the task with the formal experimental program (with mask for 40 trials).

The participants were assigned to two training groups, one with dyslexia and one control group, and completed ten sessions of TDT training within four weeks with two or three sessions per week. All reading measures and TDT were administered to the participants before and after training. In addition, a follow-up testing with all of these reading measures was conducted to the participants of two training groups two months post TDT training to investigate whether the training effect was long-lasting and maintainable.

The participants in the two non-training groups participated in the same TDT threshold and all of the same linguistic measurements as the training groups in the pre- and post-tests. However, they were not trained with the TDT. The time interval between pre- and post-tests was the same as the training groups.

### Data analysis

We first investigated whether there was difference in the TDT thresholds in the pre-test between the dyslexia and control groups. Next, the TDT learning effect on TDT processing was estimated. Then, we compared the slopes of the learning curves between the two training groups. Finally, ANOVAs were conducted for the TDT thresholds and the two reading measures, reading fluency and vocabulary in the test sessions, to examine whether the TDT training effects were significant and whether the TDT training affects the performance in the vocabulary test and the reading fluency test. The factors were Participant (dyslexia vs. control; between-subjects factor), Training Group (training vs. non-training, between-subjects factor) and Testing Time (pre- vs. post-test; within-subjects factor).

All of the ANOVAs were conducted with SPSS software. If any significant interactions related to Training Group were found, the subsequent simple effects were analyzed. In all of the analyses, in cases of sphericity violations, the significance levels of the F ratios were adjusted by the Greenhouse-Geisser correction.

## Results

### The TDT performance in the pre-test

Before training, the TDT threshold in the pre-test for the dyslexia group (584.89±59.75 ms) was significantly higher than that of control group (414.08±59.75 ms, t_34_ = −2.063, p<.048). Moreover, we compared the performance without mask in the practice. There were no significant differences in the central letter task between children with dyslexia and control children (accuracy: 94.31% vs. 95.83%; t_34_ = 0.954, p>0.351). However, when they were asked to discriminate both the central letter and peripheral stimuli simultaneously, children with dyslexia exhibited much lower accuracy than control children (75.97% vs. 89.44%; t_34_ = 3.903, p<0.001).

The “training”’ and “non-training” groups had similar pre-test TDT thresholds (t_34_ = 0.771, p>.446), therefore the observers in the training and non-training groups were homogeneous in the behavioral dimension before the training.

### The effect of TDT training on TDT

**Figure 3:**
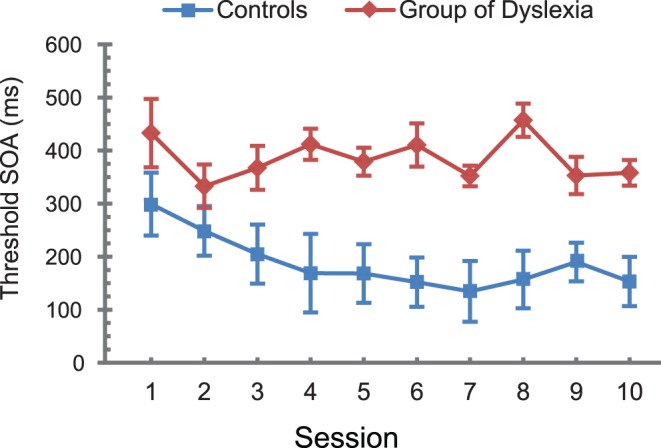
The perceptual learning curves for two training groups of children. The error bars represent standard errors.

The TDT threshold SOAs for the two training groups of children in 10 sessions were averaged separately. Learning curves depict the learning progress of the two training groups of children (Figure 3).

A repeated-measures ANOVA of 2 Participants (Controls vs. Dyslexia)×2 Training Group (Training vs. Non-training)×2 Testing Time (Post- vs. Pre-test) was conducted to estimate the training effects of TDT. The main effect of Training Group was significant (F_1,32_ = 5.09, p<.05), suggesting that the training group had a lower TDT threshold SOA (347.61±44.09 ms) than the non-training group (493.72±45.79 ms). The main effect of Participants was also significant (F_1,32_ = 6.58, p<.05), suggesting that the dyslexia group (503.72±42.10 ms) had a higher threshold SOA than the control group (337.61±43.32 ms). The main effect of testing time was also significant (F_1,32_ = 29.99, p<.0001), suggesting that the threshold SOA for post-test (341.84±27.79 ms) was significantly lower than that of pre-test (499.49±42.25 ms).

**Figure 4:**
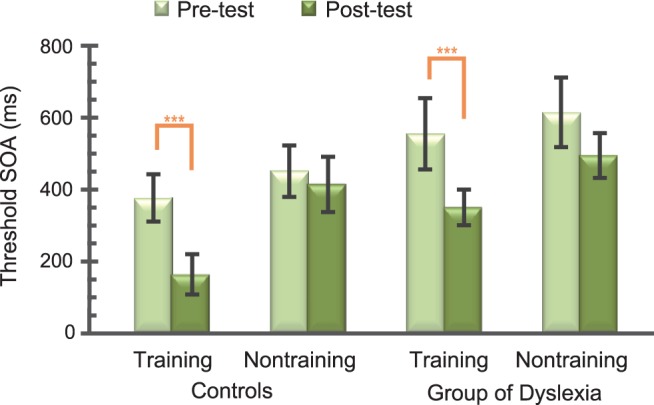
The threshold SOA (mean ± standard error) of TDT performance in the pre- and post- test.

The interaction between Training Group and Testing Time was significant (F_1,32_ = 6.78, p<.05). The post-hoc simple effect analyses showed that the threshold SOA of post-test (229.28±39.29 ms) was significantly lower than pre-test (465.93±59.75 ms) in the training group (p<.0001, Bonferroni’s correction), but not in the non-training group (p>.05, Figure 4). In addition, the three-way interaction of Testing time×Participant×Training group was not significant (F_1,32_ = 0.447, p>.507). All of these results indicate that both the children with dyslexia and the control group could attain perceptual learning.

However, further analysis of the learning curves (Figure 3) show that there is a difference in the learning speed between the two training groups. We fitted the learning curve with a logarithmic function (y = −a×ln(x)+b) for each child. The “y” is the threshold SOA in each session, the “x” is the session number. The “a” indicates the slope of the learning curve. The results show that the slope of learning curve for the dyslexia group is marginally smaller than that of the control group (62.2±69.6 vs. 7.4±42.8; t_16_ = 2.012, p = 0.061), indicating that the learning speed for the control group is faster than that of dyslexia group. Secondly, we calculated the averaged vertical distance between the threshold SOA and the logarithmic function across all of the training sessions. The averaged distance of the dyslexia group was significantly larger than that of the control group (73.7±23.4 vs. 45.4±19.2; t_16_ = −2.798, p<0.014), indicating that the threshold SOA for the group with dyslexia had much more fluctuation than that of control group during the training.

### The effect of TDT training on reading measures

**Figure 5:**
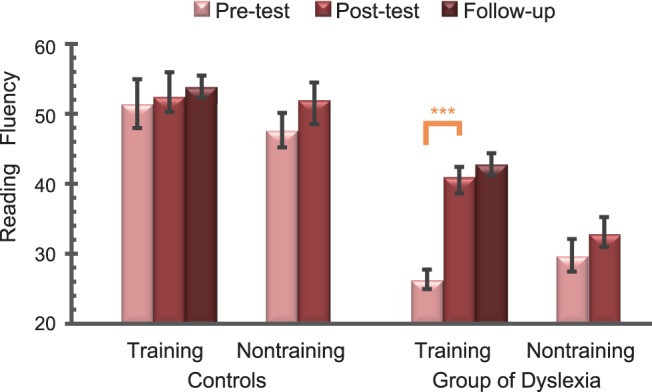
The reading fluency performance (mean ± standard error) in the pre-, post- and follow-up test.

The same repeated-measures ANOVA of 2 Participants (Controls vs. Dyslexia)×2 Training Group (Training vs. Non-training)×2 Testing Time (Post- vs. Pre-test) was conducted to estimate the training effects of perceptual training on reading measures. Reading fluency performance was enhanced in the post-test compared to the pre-test in the dyslexia training group. Specifically, the main effect of Testing Time was significant (F_1,32_ = 16.3, p<.001). The interaction of Participants and Testing Time was also significant (F_1,32_ = 4.73, p<.05). Simple effect analyses indicate that the performance of reading fluency was significantly improved in the post-test compared to that in the pre-test for the participants with dyslexia (p<.001, Bonferroni’s correction), whereas this comparison was not significant in control group (p>.1). The interaction of Testing Time, Participants, and Training Group was also significant (F_1,32_ = 6.77, p<.05). Simple effect analyses revealed that reading fluency performance in the post-test (41.00±2.57) significantly outperformed that of the pre-test (26.33±2.53) for the dyslexia training group (p<.0001, Bonferroni’s correction). However, this comparison did not reach significance in the non-training group of children with dyslexia or in the control groups (p>.1, Bonferroni’s correction, Figure 5). After 2 months, the performance improvement of reading fluency in the training group of dyslexia was still maintained, as revealed by a non-significant paired two-tailed t-test between the post-test and the follow-up test (t_8_ = 1.11, p>.1).

The same repeated-measures ANOVAs of 2 Participants (Controls vs. Dyslexia)×2 Training Group (Training vs. Non-training)×2 Testing Time (Post- vs. Pre-test) was also conducted to estimate the training effects of perceptual training on vocabulary. However, neither the main effect of Training Group nor its interactions with other factors were significant, suggesting that the TDT training did not affect the performance of vocabulary.

## Discussion

The focus of this study was to examine whether visual perceptual training with an individually adaptive visual texture discrimination task could benefit reading performance in developmental dyslexia. Both the children with dyslexia and control children decreased TDT threshold SOAs from TDT training, but the children with dyslexia had higher threshold SOAs and slower learning speed, further confirming that children with dyslexia indeed have deficits in visual perceptual processing and learning. More importantly, reading fluency performance was significantly enhanced only in the dyslexia training group, and this enhancement could be maintained up to two months post TDT training. These results suggest that visual perceptual training might have a positive effect in improving performance of reading fluency.

The enhanced reading fluency performance can be explained in three ways. First, temporal processing is one of the possible causes that underlie the training effect. A major study reported the temporal processing deficit in people with dyslexia [34]. In the present TDT task, the participants had to make judgments about the orientation of the central letter and the peripheral target bars in the short time span of SOA between stimuli and mask. Previous research has shown that TDT learning involves temporal processing to disentangle target stimuli from mask [35], [36]. Similarly, a recent study found that TDT learning was mostly temporal learning, which is the temporal learning of target–mask separation [37]. Additionally, TDT training improved participants’ temporal processing speed and probably enabled them to separate the target and the mask rapidly within a very brief threshold SOA [38], [39]. In the current study, reading fluency is a speeded comprehension task which requires participants to analyze the visual stimuli accurately and quickly, retrieve the phonological information, and access the meaning of reading materials immediately. Therefore, TDT might enhance reading fluency performance of individuals with dyslexia by strengthening their temporal processing ability. Compared to normal readers, children with dyslexia had a deficiency in temporal processing which resulted in low efficiency in separating target and mask prior to training; they might be more sensitive to the adaptive and intensive TDT training than normal counterparts and exhibited training effects.

Second, visual perceptual training possibly enlarged and strengthened the readers’ visual span and improved their reading speed through training their peripheral visual field. This interpretation is compatible with the findings from studies in people with amblyopia [21], in normally sighted young adults [40]–[42], and even in people with central vision loss [43]. In our study, even without the mask, children with dyslexia exhibited more difficulty than control children when they were asked to process central letter and peripheral stimuli simultaneously [44]. This result suggests that, at least, part of their deficit in TDT learning is not related to temporal processing. The peripheral field in individuals with dyslexia is possibly weak and therefore had difficulty in processing the visual attributes and forming representations of stimuli presented in the peripheral field [45], and visual perceptual training might be a potential effective treatment for these individuals with dyslexia.

The third explanation for these results involves spatial attention. Previous reports have suggested that the developmental dyslexia had attention deficit [46]–[48], and there may be a causal link between visual spatial attention and reading acquisition [49], and reading speed was improved through strengthening attention abilities [19]. Meanwhile, our recent ERP studies found that visual perceptual learning also involved spatial attention regulation [50], [51], and TDT training might also improve reading fluency performance by enhancing their spatial attention ability. Further study will be necessary to clarify the underlying mechanisms.

TDT training improves reading fluency in the children with dyslexia, but not the performance of vocabulary. Previous findings have suggested that visual processing in the peripheral visual field, temporal processing, and visual spatial attention might be the potential processes involved in perceptual learning [37], [43], [50]. Meanwhile, as stated earlier, reading fluency mainly targeted reading speed which demanded a certain amount of ability in temporal processing and spatial attention. Additionally, visual processing in the peripheral visual field was critically important in continuous text reading [39]. In contrast, vocabulary test, as a character recognition task, was mostly involved separate character recognition and word composition, which put less demands on temporal processing, peripheral field visual processing, and spatial attention than did reading speed task, reading fluency test. That’s plausible the reason why TDT training enhanced performance in reading fluency instead of vocabulary in the children of dyslexia. In addition, it might be the ceiling effect that resulted in the unchanged reading fluency performance in normal control group.

These findings are of two-fold practical significance: first, it has implications for the early prevention or diagnosis of potential dyslexia. Commonly, dyslexia cannot be identified until the child has failed in learning to read and write, and studies show that these children rarely catch up [52]. However, because dyslexia is a constitutional impairment, if we know more about early risk signs of dyslexia, early prevention starting before they learn to read should be possible and effective. If visual perceptual processing/learning is associated with and possibly shares common mechanisms with the development of reading skills, adaptive visual perceptual learning programs/games with very basic visual stimuli prior to school years would be beneficial for preventing at-risk readers from developing into dyslexia.

Second, effective training program should be individualized and adaptive. From the findings of the current study and the previous study [23], the perceptual learning curves and learning speed have great individual differences; in order to obtain gains for each individual, the procedural arrangement and learning pace for individuals with dyslexia should be determined in accordance with their individual basic processing and learning abilities.

## Conclusions

Our results demonstrate that visual perceptual learning can significantly enhance the performance of reading fluency in Chinese-speaking children with developmental dyslexia, further suggesting that that basic visual perceptual processing/learning and reading ability in Chinese might at least partially rely on overlapping mechanisms.
